# Effects of physical activity on cognitive function among patients with diabetes in China: a nationally longitudinal study

**DOI:** 10.1186/s12889-021-10537-x

**Published:** 2021-03-11

**Authors:** Anying Bai, Liyuan Tao, Jia Huang, Jing Tao, Jue Liu

**Affiliations:** 1 Department of Epidemiology and Biostatistics, School of Public Health, Peking University, Beijing, 100191 China; 2 Research Center of Clinical Epidemiology, Peking University Third Hospital, Beijing, China; 3 College of Rehabilitation Medicine, Fujian University of Traditional Chinese Medicine; Fujian Key Laboratory of Rehabilitation Technology, Fujian University of Traditional Chinese Medicine, Fuzhou, China

**Keywords:** Diabetes, China, Episodic memory, Executive function, Longitudinal

## Abstract

**Background:**

We aimed to examine the effect of physical activity on different cognitive domains among patients with diabetes.

**Methods:**

We used two waves of data from the Chinese Health and Retirement Longitudinal Study (CHARLS, 2013–2015), a nationally representative dataset of Chinese population aged over 45. Total physical activity scores were calculated based on the International Physical Activity Questionnaire (IPAQ). Executive function and episodic memory were used as measures of cognitive function. We conducted lagged dependent variable models to explore the association between physical activity and cognitive function in full sample as well as two different age groups (45–65, ≥65). Results: 862 diabetic patients were included. We found that diabetic participants who had greater level of physical activity at baseline were associated with better episodic memory function in 2 years (*p* < 0.05). Moreover, physical activity was significantly associated with less decline in episodic memory in fully adjusted models, and the associations were stronger among patients aged 45–65 years (*p* < 0.05). No statistically significant association was found between physical activity and executive function in all age groups.

**Conclusions:**

Physical activity may prevent some of the potential decline in episodic memory in diabetic patients. Clinicians and public health departments should strengthen the promotion of physical activity and develop early screening tools among diabetic participants to prevent the progression of cognitive impairment.

## Background

Diabetes has become a major public health concern globally. Approximately 422 million people worldwide are living with diabetes, and the prevalence of it increases with age [1]. A recent study indicated that approximately 11% of the population in China has diabetes [2], ranking one of the top three countries in the world [3]. As a leading cause of mortality, diabetes is a strong risk factor for a series of complications such as cardiovascular disease, kidney disease and neuropathy, contributing to significant burden at the individual and social level. Moreover, growing evidence have suggested the associations between diabetes-related diseases and cognitive disorders [4, 5].

Cognitive disorders are a category of mental health disorders that primarily affect cognitive abilities such as learning, memory and perception [6], including delirium and dementia. As the most serious stages in the development of cognitive dysfunction [7], dementia would considerably affect the span and quality of life among adults [8]. The consequences of diabetes and dementia present substantial individual, community, and societal impact. China has the largest population of patients with dementia in the world, imposing a heavy burden on the public and health care systems [9]. Globally, various epidemiological studies and mechanism research have discovered the correlation between diabetes and cognitive dysfunction, demonstrating that diabetic patients have lower cognitive function than healthy individuals while having a higher risk of cognitive disorders and declines diabetic patients’ quality of life and leads to severe behavior disorder [10–17]. Thus, effective interventions to prevent or reduce cognitive impairment is crucial to better care of diabetic patients. Previous research reported that the presence of diabetes was associated with an elevated risk for vascular brain damage and neurodegenerative changes [10], and may accelerate the progression from mild stage of cognitive disorder to dementia [3].

Physical activity (PA) is defined as bodily movement produced by skeletal muscles that results in energy expenditure [18]. A growing body of research suggested that greater levels of physical activity could positively influence cognitive function across the lifespan, such as simple reaction time, response accuracy and working memory, and reduce the risk of cognitive decline in adult population [19–21]. A recent meta-analysis of five randomized control trials and cohort studies involving 2581 patients with diabetes showed that physical activity was beneficial to improving cognition in patients with diabetes in studies of follow-up time less than 1 year (10.1002/dmrr.3443). However, the long-term effect with follow-up time over 1 year needs to be explored in future studies (10.1002/dmrr.3443). By contrast, recent RCTs have shown no benefit for exercise in cognitively healthy older adults [22, 23]. This contradictory evidence might be due to the fact that the parameters of PA, measurement of cognition and quality of study design were various [19]. Besides, most observational data came from western countries, and similar research among diabetic individuals in China remains scarce. Since the differences in social and cultural backgrounds, lifestyle and environmental factors may result in different implications on the diabetic-related cognitive impairment, it is necessary to explore preventive strategies among people at high risk to reduce the current and future burden of dementia in different countries.

Therefore, the primary aim of this study was to examine the effect of physical activity on cognitive function among middle-aged and older diabetic adults in China, using a 2-wave longitudinal national representative data. Besides, through the subgroup analysis, we identified whether the impact differs among various age groups. This study may be useful in helping government of China and other developing countries to improve the mental health status and quality of life among middle-aged and older diabetic populations by having a clear guidance of proceeding fitness programs.

## Methods

### Study population

**Fig. 1:**
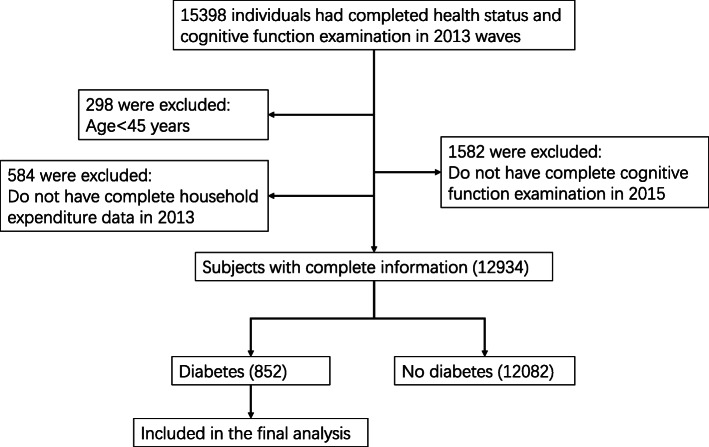
Flow diagram of the study population in the project

We used 2 waves of data from the China Health and Retirement Longitudinal Study (CHARLS, 2013–2015), which was publicly available at http://charls.pku.edu.cn. CHARLS was a nationally representative survey involved participants aged 45 years or older and their spouses, which included assessments of social, economic, and health circumstances of community-residents. Conducted between June 2011 and March 2012, the national baseline survey included 17,707 individuals from 10,287 households living in 450 villages or urban. All participants were surveyed through face-to-face household interviews, and will be periodically re-surveyed every 2 years using largely the same procedures as the baseline. From 17,707 individuals, 15,398 individuals had completed health status and cognitive function examination data in 2013, 298 of them were firstly excluded because they were younger than 45 years old, then 584 were excluded because they did not have complete household expenditure data. Furthermore, 1582 who did not have complete cognitive function examination in 2015 and subsequently 12,082 who were not diagnosed as diabetes were excluded. Eventually, 852 diabetic participants with complete information were included in the current study. The current study is a secondary analysis of the de-identified China Health and Retirement Longitudinal Study (CHARLS) public data. The original CHARLS was approved by the Ethical Review Committee of Peking University (IRB00001052–11015), and all participants signed the informed consent at the time of participation. Our research has been performed in accordance with the Declaration of Helsinki. More details on the inclusion process of studied population were provided in Fig. 1.

### Measurement of physical activity

We constructed the variable of PA scores based on the International Physical Activity Questionnaire (IPAQ), including activities for exercise, entertainment, job demand, and other purposes [24]. General questions about physical activity use in the “health status and functioning” section of CHARLS questionnaire included the amount of time a person spent on different types of physical activities (vigorous activities, moderate activities, and walking for at least 10 min continuously) in a usual week. Vigorous activities referred to the activities which made participants breathe much harder than normal, including heavy lifting, digging, plowing, aerobics fast bicycling, and cycling with a heavy load. Moderate activities included carrying light loads, bicycling at a regular pace, or mopping the floor. Walking at work and home, walking to travel from place to place, and walking for recreation, sport, exercise, or leisure were also reported. According to the responses, we indexed the amount of PA in 1 day as 1 (< 0.5 h); 2 (0.5–2 h); 3 (2–4 h); and 4 (> 4 h). The weekly PA duration score was calculated by multiplying the number of days and the daily PA duration index for each activity. Finally, we generated the variable of PA score using metabolic equivalent (MET) multipliers as follows: PA score = 8.0 × total vigorous activity weekly duration score + 4.0 × total moderate activity weekly duration score + 3.3 × total walking weekly duration score [25].

### Measurement of cognitive function

Based on similar concepts in HRS [26], two dimensions of cognitive function were evaluated by a composite battery of cognitive tests in CHARLS, including episodic memory and executive function.

Episodic memory was a necessary component of reasoning in many cognitive dimensions. It was accessed through the immediate recall and delayed recall in a word recall test [10]. Participants were requested to immediately repeat the 10 Chinese words after these were read to them by interviewers. Then after questions concerning depression status (approximately 4 to 10 min), participants were again asked to recall as many of the original words as possible. A successful recall was coded as 1; otherwise as 0. Scores ranged from 0 to 10 for both immediate and delayed recall. An evaluated episodic memory was expressed as the means of scores in the immediate and delayed word recall.

The second cognitive measure was based on the Telephone Interview of Cognitive Status (TICS), to capture the executive function of individual cognitive function. The TICS was an 11-item screening test including serial subtractions of 7 from 100 (up to 5 times), date (month, day, and year and season), the day of the week, and the ability to redraw a picture shown to him/her [27]. The sum of correct answers (range 0 to 11) was calculated to measure the executive function of a person.

### Definition of diabetes status

The CHARLS survey asked two questions about diabetes: “Have you been diagnosed with diabetes or high blood sugar by a doctor?” and “Are you now taking any of the following treatments to treat or control your diabetes?” Diabetes was defined as (1) a self-reported previous diagnosis by health care professionals or currently taking medicine to treat or control diabetes (2) fastng plasma glucose (FPG) level of 126 mg/dL and/or glycated hemoglobin (HbA1c) level of 6.5% or higher. The cut-off points for diagnosis of diabetes were based on current recommendations from the American Diabetes Association [28].

### Covariates

The covariates included in the analysis were sociodemographic characteristics and health status related to cognitive function. Sociodemographic variables included age, gender, resident areas (urban or rural), education levels (illiterate, primary education, secondary or above), marital status (currently married and/not living with spouse, divorced/widowed/or never married) and household expenditure. Smoking status was categorized as never, former, or current smoker. Health status include a history of stroke and heart disease (including heart attack, coronary heart disease, angina, congestive heart failure, or other heart problems), chronic diseases (including hypertension) and the Center for Epidemiologic Studies Depression (CES-D) Scale, which was used to measure depressive symptoms. The baseline chronic disease of hypertension was classified as three types based on self-reported conditions on whether the participants were being treated: having hypertension with treatment, having hypertension without treatment and not having hypertension. Each of the 4-option response to the item in CER-D Scale short form was scored ranging from 0 to 3, and the total score is the sum of points for all 10 items. A total score of 12 or higher indicated the presence of clinical depression.

### Statistical analysis

Characteristics of the study population were described using means and standard deviations for continuous data, and numbers and percentages were used for categorical data. To infer associations and avoid reversed causality, we used lagged dependent variable (LDV) models with ordinary least squares estimation to examine the relationships between baseline physical activity and cognitive function after 2 years. LDV models had superiority in analyzing the effects of predictor variables on an outcome with 2-wave panel data and controlling for the influence of time-invariant variables meanwhile [29, 30]. It adjusted for baseline cognitive conditions for all participants, therefore provided more robust estimates of the effects of independent variables [31]. Additionally, we used progression of regressions. For each outcome variable, we added in different cluster of variables in different steps. First, unadjusted models were analyzed on the associations between physical activity and cognitive function. Second, we added in sociodemographic variables including age, gender, living areas, education levels, marital status and household expenditure to model 2. In model 3, baseline cognition scores were added. Additionally, we adjusted for hypertension status and depression level in model 4. In the subgroup analysis, study population were classified into two age groups (45–65, ≥65) in order to evaluate whether the relationship between physical activity and cognition was age-dependent among diabetic participants. Robust standard errors were used to adjust for heteroskedasticity. We presented beta coefficient and *p* value for each variable in models. All reported *p* values were two-tailed, with a significance level of 0.05. All analyses were performed using STATA software (version 14.0; Stata Corp LP. TX).

## Results

### Subjects characteristics

Table 1 provides a descriptive summary of the variables for full study diabetic patients (*n* = 856). Participants were 61.41 years old on average, with more than half of them were female (55.7%) and living in urban area (53.29%). The average scores of cognitive function was 3. 63 ± 1.70 for episodic memory and 8.28 ± 2.74 for executive function in 2013, and the average scores of both cognitive functions decreased in 2015.

**Table 1:** Sociodemographic and health characteristics of participants with diabetes

Characteristics	Overall (***N*** = 852)
**Baseline Total Physical Activity Score, Mean (SD)**	32.84 (73.90)
**Age, Mean (SD)**	61.41 (8.61)
**Gender, No. (%)**	
**Female**	485 (56.7%)
**Male**	367 (42.9%)
**Area, No. (%)**	
**Urban**	454 (53.29%)
**Rural**	398 (46.71%)
**Education, No. (%)**	
**Illiterate**	323 (37.91%)
**Primary education**	202 (23.71%)
**Secondary or above**	327 (38.38%)
**Marital Status, No. (%)**	
**Married**	756 (88.73%)
**Unmarried/divorced**	96 (11.27%)
**Annual Household Expenditure, Mean (SD), yuan**^**a**^	20,627.30 (27,335.18)
**Executive Function Scores, Mean (SD)**	7.51 (3.26)
**Episodic Memory Scores**^**d**^**, Mean (SD)**	3.21 (1.86)
**Baseline Executive Function Scores, Mean (SD)**	8.28 (2.74)
**Baseline Episodic Memory Scores, Mean (SD)**	3.63 (1.70)
**Baseline CES-D Scores**^**e**^**, Mean (SD)**	11.10 (5.32)
**Smoking Status, No. (%)**	
**Never smoker**	790 (92.72%)
**Former smoker**	34 (3.99%)
**Current smoker**	28 (3.29%)
**History of Stroke, No. (%)**	43 (5.05%)
**History of Heart Disease, No. (%)**	196 (23.00%)
**Hypertension, No. (%)**	
**No hypertension**	470 (55.16%)
**Hypertension with treatment**	328 (38.50%)
**Hypertension without treatment**	54 (6.34%)

### Association between Total physical activity and cognitive function

Table 2 summarizes the unadjusted and adjusted results of linear regression models between physical activity and episodic memory scores as well as executive function scores among the patients. Diabetic patients who have higher physical activity scores at baseline were associated with better performance in episodic memory in 2 years in the unadjusted model (β = 0.0015, *p* < 0.1). After adjusting for sociodemographic characteristics, baseline health status and cognitive function, the association between total physical activity scores at baseline and episodic memory in 2 years was still significant (β = 0.0015, *p* < 0.05). By contrast, the effect of physical activity on executive function was not significant in all models.

**Table 2:** Associations between Physical Activity and Cognitive function by lagged dependent variable models

Variable(***N*** = 852)	Executive Function	Executive Function	Executive Function	Executive Function
**β(p)**	**Model 1**	**Model 2**	**Model 3**	**Model 4**
**Total Physical Activity**	−0.0004(0.0013)	0.0009(0.0010)	−0.0004(0.0008)	− 0.0003(0.0009)
**Memory base**			0.1375**(0.0547)	0.1369**(0.0547)
**Mental base**			0.5697***(0.0393)	0.5603***(0.0403)
***R***^**2**^	0	0.28	0.458	0.462
**F**	0.0954	38.4167	82.2564	48.0593
**Variable(N = 852)**	**Episodic Memory**	**Episodic Memory**	**Episodic Memory**	**Episodic Memory**
**β(p)**	**Model 1**	**Model 2**	**Model 3**	**Model 4**
**Total Physical Activity**	0.0014*(0.0008)	0.0018**(0.0008)	0.0015**(0.0007)	0.0015**(0.0007)
**Memory base**			0.3740***(0.0358)	0.3726***(0.0356)
**Mental base**			0.1159***(0.0234)	0.1124***(0.0245)
***R***^**2**^	0.003	0.221	0.369	0.373
**F**	2.9595	30.4477	56.1891	33.0257

Model 1: Unadjusted; Model 2: Adjusted for age, gender, living areas, education levels, marital status and household expenditure; Model 3: Further adjusted for baseline cognitive function; Model 4: Further adjusted for smoking status, history of stroke, heart problem, hypertension status and depression level.

Source: CHARLS 2011,2013,2015 waves

* *p* < 0.1, ** *p* < 0.05, *** *p* < 0.01

In subgroup analysis, after adjusting for all potential confounders, physical activity (β = 0.0016, *p* < 0.05) was significantly associated with episodic memory among the age group of 45–64, while there was no significant relationship between physical activity scores and executive function in all age groups (Table 3).

**Table 3:** Associations between Physical Activity and Cognitive function Stratified by age group

	**45 < age < 65**	**age > 65**
**Variable β(p)**	**Episodic Memory(*N*** **= 557)**	**Episodic Memory(*N*** **= 295)**
**Total Physical Activity**	0.0016**(0.0008)	0.0011(0.0017)
**Memory base**	0.3772***(0.0453)	0.3603***(0.0622)
**Mental base**	0.1133***(0.0325)	0.0992**(0.0386)
***R***^**2**^	0.328	0.401
**F**	17.3350	13.8767
	**45 < age < 65**	**age > 65**
**Variable β(p)**	**Executive Function(N = 557)**	**Executive Function(N = 295)**
**Total Physical Activity**	−0.0001 (0.0010)	−0.0010 (0.0023)
**Memory base**	0.1290**(0.0656)	0.1613*(0.0962)
**Mental base**	0.6081***(0.0500)	0.4629***(0.0649)
***R***^**2**^	0.428	0.530
**F**	25.8365	27.6147

Model adjusted for age, gender, living areas, education levels, marital status, household expenditure, baseline cognitive function, smoking status, history of stroke, heart problem, hypertension status and depression level.

Source: CHARLS 2011,2013,2015 waves

* *p* < 0.1, ** *p* < 0.05, *** *p* < 0.01

### Association between Total physical activity and decline of cognitive function

Table 4 shows the results of associations between physical activity at baseline and the decline of cognitive function in 2 years. Physical activity was significantly associated with less decline in episodic memory in crude (β = 0.0015, *p* < 0.1) and fully adjusted (β = 0.0015, *p* < 0.05) models. Nevertheless, there was no significant relationship between physical activity and decline of executive function (*p* > 0.05).

**Table 4:** Associations between Physical Activity and the Decline of Cognitive function

**Variable(*N*** **= 852)**	**Executive Function**	**Executive Function**	**Executive Function**	**Executive Function**
**β(p)**	**Model 1**	**Model 2**	**Model 3**	**Model 4**
**Total Physical Activity**	−0.0014(0.0010)	− 0.0013(0.0010)	− 0.0004(0.0008)	− 0.0003(0.0009)
**Memory base**			0.1375**(0.0547)	0.1369**(0.0547)
**Mental base**			−0.4303***(0.0393)	−0.4397***(0.0403)
***R***^**2**^	0.002	0.015	0.140	0.146
**F**	2.0774	1.6041	13.0550	8.0709
**Variable(N = 852)**	**Episodic Memory**	**Episodic Memory**	**Episodic Memory**	**Episodic Memory**
**β(p)**	**Model 1**	**Model 2**	**Model 3**	**Model 4**
**Total Physical Activity**	0.0014*(0.0008)	0.0015*(0.0008)	0.0015**(0.0007)	0.0015**(0.0007)
**Memory base**			−0.6260***(0.0358)	−0.6274***(0.0356)
**Mental base**			0.1159***(0.0234)	0.1124***(0.0245)
***R***^**2**^	0.004	0.015	0.282	0.287
**F**	3.0448	1.6617	34.8610	21.2585

Model adjusted for age, gender, living areas, education levels, marital status, household expenditure, baseline cognitive function, smoking status, history of stroke, heart problem, hypertension status and depression level.

Source: CHARLS 2011,2013,2015 waves

* *p* < 0.1, ** *p* < 0.05, *** *p* < 0.01

## Discussion

Using a 2 year-wave nationally representative dataset of a 2 years follow-up, we examined the effect of physical activity on different cognitive subdomains among diabetic participants above 45 years old in China. Additionally, we explored this relationship in different age groups and controlled for the effect of age, gender, living area, marital status, education, expenditure, baseline cognitive function, depression and chronic conditions. Results displayed that diabetic participants who had higher level of physical activity were associated with less decline in episodic memory function in 2 years. The associations were also significant among diabetic participants aged 45 ~ 65 years old. Our findings added to the evidence of long-term effect of physical activity on improving cognition function in patients with diabetes in a longitudinal study of two-years follow-up.

Our study found that in all the 862 diabetic participants, physical activity scores were consistently associated with episodic memory function in crude and fully adjusted analysis. Additionally, greater level of physical activity was also significantly associated with less decline in episodic memory in a 2-year period. Previous evidence on this relationship was limited and controversial. The Lifestyle Interventions and Independence for Elders (LIFE) trial enrolling adults aged 70–89 years who were sedentary reported better global cognitive function and delayed memory among diabetic individuals after physical activity intervention [32], while two cohort studies indicated little overall associations between physical activity and higher mental function measured by Mini-Mental State Exam (MMSE) among diabetic patients [33, 34]. Although recent reviews also reported the positive effect of physical activity on cognition among diabetic population, none of them have statistically quantified the findings into a numerical estimate of effect, and the conclusions were controversial [35–37]. The inconsistency might be due to the difference in characteristics of participants, types and duration of physical activity, and measures of cognition.

Moreover, after exploring the associations between physical activity and different domain of cognitive function, we observed evidence of benefit for episodic memory rather than executive function. In CHARLS questionnaires, episodic memory mostly focused on memorizing ability, while executive function involved other mental abilities, such as reading and calculating. Previous studies showed that physical activity was linked with reduced brain atrophy, which might support the decline in executive control process and memory function among older adults [38–40], and Baker, et al. also found that physical activity was associated with improvement in executive function, but not memory in 28 newly-diagnosed adults (aged 57 ~ 83 years old) [41]. One cross-sectional study among middle-aged and old-aged participants in China reported that untreated diabetes or elevated HbA1c concentrations had a larger effect on participants’ episodic memory function rather than executive function [10], which might partly explain our results that the effect of intervention on physical activity was mostly on episodic memory function. Differences among the health conditions of subjects, study period, included physical exercise and measurement of cognition may account for the inconsistent findings.

To assess the different effect of age in the associations, we classified the full sample into two groups (45 ~ 64, ≥65), and conducted multiple regression models separately while controlling the same covariates as in full sample analysis. Consequently, the association between physical activity and episodic memory could only be seen among middle-aged adults (aged 45 ~ 65 years) and the associations was stronger than the full sample. We observed no significant relationship among older adults aged ≥65 years. Previous research reported inconclusive findings on the effect of physical activity on cognitive function in older adults: a prospective study of rural elderly people aged over 65 years found that all levels of exercise participation could prevent the decline of cognition measured by MMSE over a 2-year interval [42], and the meta-analyses also reported that physical exercise interventions (eg. resistance training and taichi) were effective at improving the cognitive function of older adults. However, another research in people older than 50 demonstrated little benefit of exercise (eg. resistance training and taichi) on cognitive function [43]. Since the above studies were mostly conducted in healthy older adults with interventions restricted to only one type of exercise, the applicability of these findings to older adults with metabolic illness is less well defined. By contrast, we measured physical activity based on the metabolic equivalent (MET) multipliers of all types of exercise, considering the duration and frequency of different exercise at the same time. Therefore, our conclusions might be more robust compared with previous studies.

The possible mechanism linking physical activity and cognition among diabetic participants might primarily through biomarkers such as elevated neurotrophin levels, improved vascularization, better blood glucose other signaling pathways that could benefit brain function [44, 45]. Among diabetic patients, it is found that energy expenditure brought by physical activity might decrease insulin resistance (IR),which contributed to cognitive impairment through a vascular mechanism [46]. Physical activity also reduced the risk of other vascular complications regardless of the intensity and type of exercise [33]. Since hyperinsulinemia is neurotoxic, it is possible that improved insulin sensitivity after physical activity would favor neurogenesis, whereby elevated cognitive function [37, 47]. Further studies are needed to explore the mechanism and pathway of physical activity on effect of reduce cognitive impairment.

Our studies have several strengths. Firstly, this might be the first longitudinal study to examine the associations between physical activity and different domains of cognitive function among middle-aged and old-aged diabetic individuals in China, which provided extra evidence for the existing literature. Secondly, we conducted the study in a nationally representative cohort of community-dwelling Chinese adults, so our findings are generalizable to middle-aged and older adults in China. Finally, stringent quality control and quality assurance measures were implemented in every stage of the CHARLS study, so the quality of current study can be guaranteed. Nevertheless, some limitations should also be acknowledged. First, we could not examine the potential mediating effects or pathway from physical activity to cognitive decline among diabetic patients in the present study, because that the treatment and biological assays (eg. FPG, HbA1c) were not included in CHARLS second wave and biomarkers (such as IR, neurotrophin levels, vascularization and signaling molecules) were not tested in CHARLS survey. Second, PA related questions were not asked to all responses because of missing data on PA in CHARLS, selection bias might exist. Moreover, since the questions about physical activity in CHARLS questionnaire only included vague amount of time a person spent on different types of physical activities in a usual week, the exact amount of METs of physical activity could not be calculated. Therefore, the participants could not be categorized as previous studies [48] and the further dose-response effect between physical activity and cognitive function could not be examined in this study. Third, since our study was conducted among Chinese population, the conclusions might not be extrapolated to other countries. Evidence from prospective cohort studies and RCT in other countries is needed to further exemplify these conclusions.

## Conclusions

To conclude, this nationally longitudinal study indicated that physical activity was linked to better performance in episodic memory function among middle-aged and old-aged diabetic individuals (45 ~ 65 years old), and physical activity may prevent some of the potential decline in cognition function over 2 years’ time. Clinicians, communities and public health departments should strengthen the promotion of physical activity among diabetic participants, and develop early screening tools to prevent the progression of cognitive impairment. Based on the current evidence, we could not confirm the causative association between physical activity and cognition function. In the future, more evidence from epidemiologic, experimental and clinical studies with larger sample sizes and extended cognitive function measurements are required to clearly identify the effect of physical activity on cognitive function.

## Data Availability

The datasets analysed during the current study was publicly available at http://charls.pku.edu.cn.

## Abbreviations

CHARLS: China Health and Retirement Longitudinal Study
PA: Physical Activity
MMSE: Mini-Mental State Exam
IPAQ: International Physical Activity Questionnaire
TICS: Telephone Interview of Cognitive Status
